# Climate, environmental, and programmatic correlates of malaria resurgence in Amhara, Ethiopia (2018–2024): a Bayesian spatiotemporal analysis

**DOI:** 10.1186/s12936-026-05847-7

**Published:** 2026-03-04

**Authors:** Mastewal Worku Lake, Mulusew Andualem Asemahagn, Kassahun Alemu Gelaye, Kindie Fentahun Muchie, Teshager Zerihun Nigussie, Hailemariam Awoke Engedaw, Muluken Azage Yenesew

**Affiliations:** 1 Department of Epidemiology and Biostatistics, School of Public Health, College of Medicine and Health Sciences, Bahir Dar University, Bahir Dar, Ethiopia; 2 Amhara Public Health Institute, Bahir Dar, Ethiopia; 3 Department of Health System and Health Economics, School of Public Health, College of Medicine and Health Sciences, Bahir Dar University, Bahir Dar, Ethiopia; 4 Department of Epidemiology and Biostatistics, Institute of Public Health, College of Medicine and Health Sciences, University of Gondar, Gondar, Ethiopia; 5 Department of Infectious Disease and Tropical Medicine, University Hospital Heidelberg, Heidelberg, Germany; 6 Department of Statistics, College of Natural and Computational Sciences, Debre Tabor University, Debre Tabor, Ethiopia; 7 Department of Internal Medicine, School of Medicine, College of Medicine and Health Sciences, Bahir Dar University, Bahir Dar, Ethiopia; 8 Department of Environmental Health, School of Public Health, College of Medicine and Health Sciences, Bahir Dar University, Bahir Dar, Ethiopia

**Keywords:** Malaria resurgence, Climate variability, Bayesian spatiotemporal modelling, INLA, BYM2, Variance decomposition, Amhara, Ethiopia

## Abstract

**Background:**

After substantial progress in malaria control, Ethiopia's Amhara Region experienced a marked resurgence since 2018. The relative contributions of climate variability, environmental context, intervention coverage, and unmeasured factors to this resurgence remain inadequately quantified. This study used a Bayesian spatiotemporal framework to estimate factor associations with malaria incidence, decompose spatial versus temporal climate effects, and identify persistent hotspots.

**Methods:**

We conducted an ecological district-level panel analysis of 13,944 district-month observations from 166 districts (January 2018–December 2024). Monthly confirmed malaria counts (total, *Plasmodium falciparum*, *P. vivax*) were modelled using Bayesian hierarchical negative binomial regression with BYM2 spatial and AR(1) temporal random effects, fitted with integrated nested Laplace approximation. Covariates included lagged rainfall, temperature, NDVI, elevation, and programmatic indicators (ITN ownership, IRS protection, and larval source management [LSM] intensity). Climate covariates were decomposed into between-district (spatial) means and within-district (temporal) deviations. Sensitivity analyses included alternative IRS protection windows and district fixed-effects models.

**Results:**

A total of 5,746,571 confirmed cases were reported (64.3% *P. falciparum*, 35.7% *P. vivax*). Mean monthly incidence increased 5.5-fold from 1.19 per 1,000 (2018) to 6.53 per 1,000 (2024), while regional mean maximum temperature showed a small declining trend over the period. In fully adjusted models, higher lagged maximum temperature and rainfall were associated with higher incidence, and elevation was protective. IRS protection, higher ITN ownership, and higher LSM intensity were each associated with lower incidence; effect directions were consistent in within-district sensitivity analyses, although residual confounding and measurement error cannot be excluded. Climate–incidence associations were predominantly spatial (between-district) rather than temporal (within-district), suggesting that geographic ecological suitability explains much of the spatial patterning, rather than temporal warming trends explaining the resurgence. Districts with persistently elevated residual spatial risk (exceedance probability of residual RR > 1.25) clustered in low-elevation western border areas.

**Conclusions:**

Malaria resurgence in Amhara (2018–2024) occurred alongside strong spatial climatic and elevational gradients and was not consistent with a temporal warming-driven explanation at the regional scale. Remaining unexplained spatiotemporal variation highlights the likely importance of unmeasured drivers (e.g., conflict-related service disruption, vector/insecticide resistance dynamics, and population mobility). Climate-informed, spatially targeted intervention packages prioritizing districts with persistently high residual risk are warranted.

**Supplementary Information:**

The online version contains supplementary material available at 10.1186/s12936-026-05847-7.

## Introduction

Malaria remains a major global health problem, with an estimated 282 million cases and 610,000 deaths in 2024. About 94% of cases occurred in the WHO African Region [1]. Over the past two decades, large-scale investments in vector control (insecticide-treated nets (ITNs), indoor residual spraying (IRS), diagnostics, and artemisinin-based therapies have led to historic declines in malaria morbidity and mortality [2]. However, this progress has stalled, and many regions across sub-Saharan Africa are now witnessing a resurgence, threatening to reverse hard-won gains and challenging global elimination targets [3, 4].

Ethiopia exemplifies malaria’s complexity at the margins of stable endemicity, where transmission is inherently unstable and heterogeneous [5, 6]. Approximately 69% of the population lives in malaria‑risk areas, with altitude, rainfall, and land use modulating risk strongly [7]. Amhara Region, home to over 22 million people, is ecologically diverse, with elevations ranging from < 1,000 m in the Tekeze River valley to > 4,000 m in the Simien Mountains [8]. This topographic variation creates sharp ecological gradients that shape vector distributions. Transmission is seasonal, peaking after the main kiremt rains (June–September). Historically dominated by *Plasmodium falciparum*, the region has recently seen a rising contribution from *P. vivax*, which accounted for 35.7% of cases during the present study period [9].

Following a period of remarkable success in reducing national malaria incidence [6, 10], Amhara has experienced a notable resurgence. By 2024, regional malaria incidence had increased more than fivefold compared to 2018, despite reportedly high ITN ownership [11]. Transmission has expanded into highland-fringe areas, accompanied by an increasing proportion of *P. vivax* [12, 13]. This reversal threatens Ethiopia's goal of sub-national elimination and suggests that our understanding of the underlying drivers is incomplete.

The previously documented this resurgence in detail, demonstrating through Joinpoint regression and spatial clustering analyses that malaria incidence in Amhara increased more than fivefold between 2018 and 2024, with notable shifts in parasite species composition toward Plasmodium vivax and geographic expansion of transmission hotspots into highland and urban settings [14]. That descriptive epidemiological analysis established the magnitude, timing, and spatial patterns of the resurgence but did not quantify the relative contributions of climatic, environmental, and programmatic factors driving these changes. The present study builds upon this foundational work by employing a Bayesian spatiotemporal modelling framework to identify and quantify the specific determinants of the observed resurgence.

The resurgence is likely multifactorial. Climatic factors, particularly temperature and rainfall, fundamentally shape malaria transmission by influencing mosquito development, survival, and the parasite’s extrinsic incubation period [15, 16]. Climatic anomalies can create favorable conditions for vector breeding and extend the geographic range of transmission [9, 17]. P. vivax’s ability to relapse via liver-stage hypnozoites creates a persistent, difficult-to-detect parasite reservoir [18–20]. At the same time, insecticide resistance in *Anopheles arabiensis* could have reduced the impact of IRS and ITNs [21].

Climate—malaria relationships in Ethiopia have been studied extensively [9, 10, 17, 22–24], but important gaps remain. First, few analyses have used high-resolution district-level data collected over multiple years of documented resurgence with consistent case definitions and diagnostic protocols. Second, many prior studies did not jointly model climate, environmental context, and intervention coverage within a unified spatiotemporal framework that captures both spatial dependence and temporal autocorrelation. Third, Species-specific responses of *P. falciparum* and *P. vivax* to climatic and programmatic factors in highland fringe ecologies remain poorly quantified, and finally, decomposition of spatial versus temporal climate effects has rarely been undertaken, leading to ambiguity about whether observed climate–incidence associations reflect spatial ecology or temporal climate change.

This study aimed to identify and quantify associations of climatic, environmental, and programmatic factors with total and species-specific malaria incidence in the Amhara Region, Ethiopia, from 2018 to 2024, using a Bayesian spatiotemporal framework. Decompose climate–incidence associations into spatial (between-district) and temporal (within-district) components to distinguish spatial ecology from temporal climate trends. Quantify the proportion of variance in malaria incidence explained by measured covariates versus unmeasured spatiotemporal factors. Identify persistent hotspots as priority areas for intensified, climate-informed control, consistent with the WHO "High burden to high impact" approach [25]. We hypothesized that higher temperature and rainfall would be associated with increased incidence, that *P.* *falciparum* incidence would be more sensitive to contemporaneous climate than *P. vivax* incidence, that low-elevation western border districts would remain hotspots after adjustment for measured covariates and A substantial proportion of variance would remain unexplained by measured climate and intervention covariates, consistent with contributions from unmeasured factors including conflict, *An. stephensi* presence, insecticide resistance intensity, and health system performance.

### Conceptual framework

Climatic variables (rainfall, temperature, humidity, and seasonal patterns) influence environmental conditions such as surface water and vegetation, which in turn shape mosquito breeding site availability, survival, gonotrophic cycle duration, and sporogony rate. These vector dynamics determine entomological inoculation rates and, ultimately, clinical incidence. Vector control interventions (ITNs, IRS, and LSM) and health system performance (e.g., access to care, surveillance responsiveness, programme fatigue) modify these pathways by reducing human–vector contact and parasite reservoirs. Reactive interventions may be triggered by observed surges in cases, introducing feedback loops.

**Fig. 1:**
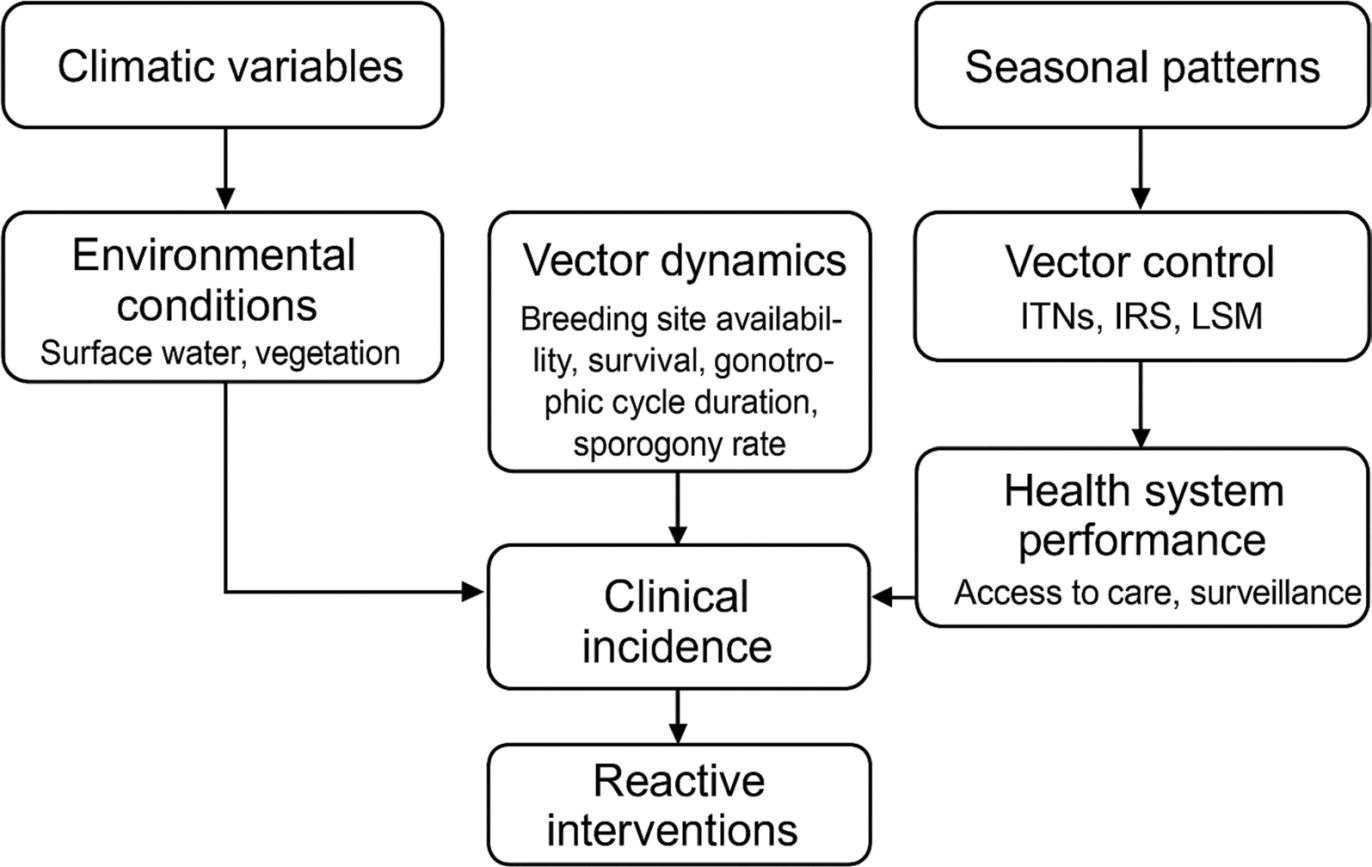
Conceptual framework linking climate, environment, vector dynamics, transmission, and interventions

This conceptual framework (Fig. 1) guided the selection of climate, environmental, and intervention covariates, the choice of temporal lags, and the inclusion of both spatial and temporal random effects in the statistical models.

## Methods

### Study design and period

**Fig. 2:**
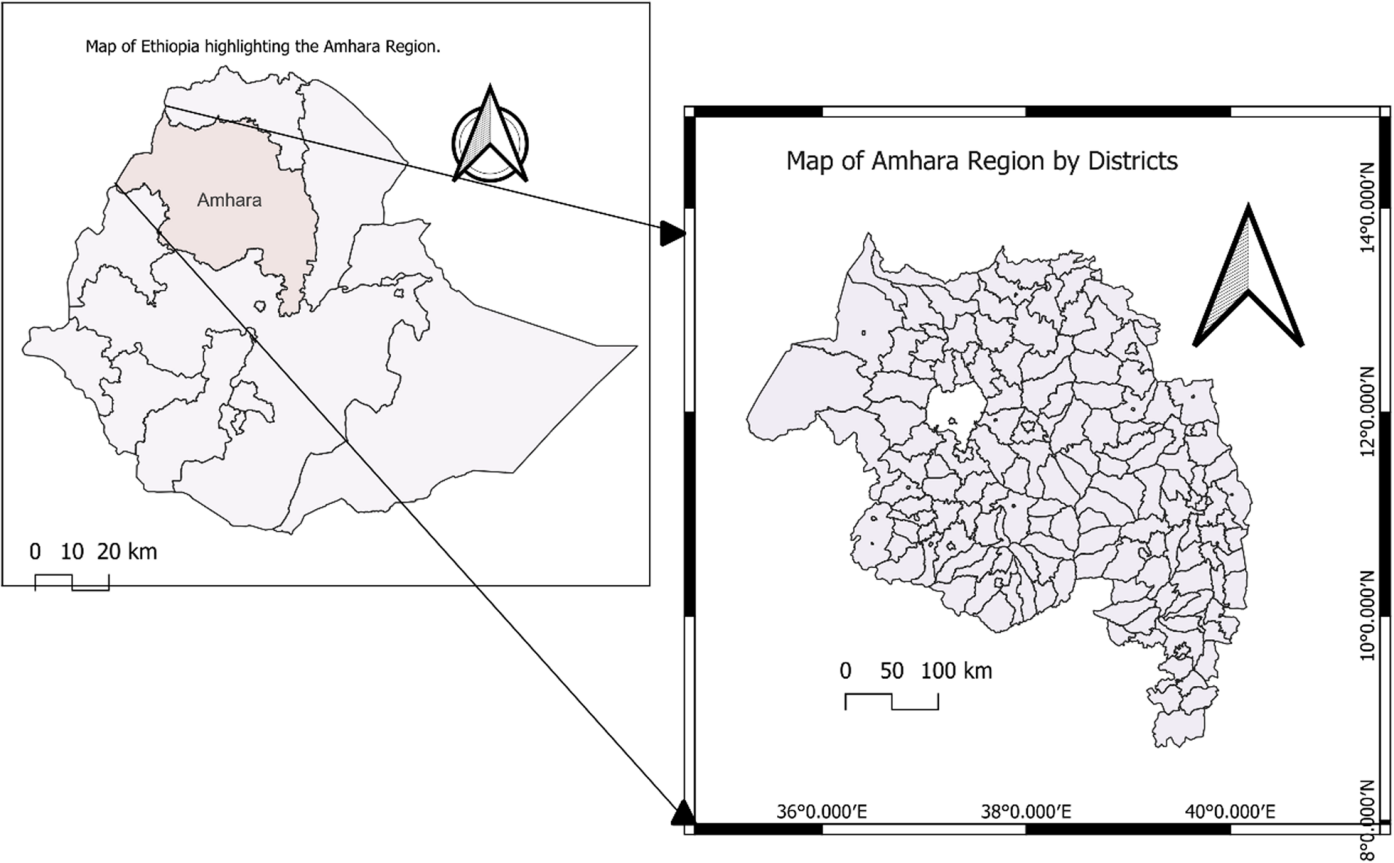
Map of Amhara Region showing study districts included in the analysis

We conducted a retrospective ecological, district-level time series study using monthly malaria surveillance data from 1 January 2018 to 31 December 2024 (84 months) (Fig. 2).

### Study setting

Amhara Region (9°–14°N, 36°–40°E) is located in northwestern Ethiopia and comprises 22 administrative zones subdivided into 236 districts (woredas), the primary units for health service delivery [26]. Elevation ranges from about 500 m to over 4,000 m above sea level, creating steep gradients in climate, vegetation, and malaria risk [27]. Both *P. falciparum* and *P. vivax* are endemic, with *Anopheles arabiensis* as the primary vector. Transmission is unstable and seasonal, with peaks typically following the main rainy season [28, 29]. Both *P. falciparum* and *P. vivax* are endemic in *An. arabiensis* is the principal vector, with *An. stephensi* emerging in some areas [28–30].

Multiple zones in the Amhara region have experienced armed conflict and population displacement since 2020, with documented health facility closures, commodity stockouts, and interruptions to routine malaria control activities [31]. These disruptions are not systematically captured in routine surveillance data.

Of the 236 districts, 166 were included based on consistent reporting coverage across 2018–2024. Weekly case reports were aggregated to monthly counts at the district level, yielding 13,944 district-months for analysis. These districts cover the majority of malaria-endemic areas and of the population at risk in Amhara.

### Outcomes

Outcomes were district-month counts of confirmed: (i) total malaria; (ii) *P. falciparum*; and (iii) *P. vivax*. Confirmed cases were diagnosed by microscopy or rapid diagnostic test according to national guidelines [7].

### Data sources

#### Malaria surveillance data

Monthly counts of confirmed malaria cases (total, *P. falciparum*, *P. vivax*) from January 2018 to December 2024 were obtained from the Amhara Public Health Institute (APHI) public health emergency management surveillance database. Cases were confirmed by microscopy or rapid diagnostic tests (RDTs) following national guidelines. Diagnostic protocols and case definitions remained unchanged during the study period. Data were aggregated to the district-month level for analysis.

Weekly district-level line lists were aggregated to monthly counts for analysis. Mixed infections (*P. falciparum* + *P. vivax*) represented < 1% of cases and were proportionally allocated to single-species counts based on the district-month species ratio.

#### Population denominators

Annual mid-year district population projections for 2018–2024 were obtained from the Central Statistical Agency of Ethiopia [26]. Monthly interpolation, in the absence of monthly vital registration, monthly district populations were linearly interpolated within each year, assuming constant within-year growth. Specifically, for district *i* and month *t* within year *y*:

$${E}_{it}={P}_{iy}+\left(\frac{{P}_{i,y+1}-{P}_{iy}}{12}\right)\times (t-1)$$

where ${P}_{iy}$ is the mid-year population for district *i* in year *y*. These monthly populations ${E}_{it}$ were used as log offsets in regression models.

#### Climatic and environmental covariates

Remotely sensed climatic and environmental data for January 2018–December 2024 were compiled:

- Rainfall (mm/month): Climate Hazards Group InfraRed Precipitation with Stations (CHIRPS v2.0) at 0.05° (~ 5 km) resolution [32].
- Temperature (°C): mean, minimum, and maximum 2-m air temperature from ERA5-Land reanalysis at 0.1° (~ 11 km) resolution [33].
- Relative humidity (%): monthly mean relative humidity from ERA5-Land at 0.1° resolution [33].
- Soil moisture: volumetric soil water content from ERA5-Land at 0.1° resolution.
- Vegetation: NDVI from MODIS MOD13Q1 250-m 16-day composites, aggregated to monthly means [34].
- Elevation (m): mean district elevation from the Shuttle Radar Topography Mission (SRTM) digital elevation model (30–90 m resolution) [27].

Spatial processing: all raster layers were projected to UTM Zone 37N (EPSG: 32637), clipped to Amhara Region boundaries, and resampled to a common 1-km grid using bilinear interpolation. District-level monthly means were computed using zonal statistics (area-weighted mean) in R [15–17, 22].

Continuous covariates were standardised (mean = 0, SD = 1) across all district-months so that regression coefficients represent the effect per SD increase.

#### Intervention covariates

Intervention data were obtained from the APHI Malaria Control Programme and the National Malaria Elimination and Control Programme:

- ITN ownership: annual district-level estimates of the proportion of households owning at least one ITN, derived from campaign administrative data and routine reports, were interpolated to monthly values using cubic splines.
- IRS: using district-specific IRS calendars, a monthly binary indicator was defined to specify whether a district-month fell within an assumed 6-month post-spray effectiveness window. If the IRS was completed in the month $m$, months $m$ through $m+5$ were coded as protected (= 1); all other months were coded as unprotected (= 0). This reflects the residual efficacy of pirimiphos-methyl CS used in Amhara during the study period [21, 30]. Sensitivity analyses applying 4- and 8-month windows were conducted.
- Sensitivity analyses applied alternative 4-month and 8-month windows (Supplementary Table S2). Only 21.5% of the 13,944 district-months fell within the 6-month post-spray protection window, reflecting limited geographic and temporal IRS coverage.
- Larval source management (LSM): the total surface area of water bodies treated per month (m^2^) was standardised per 1,000 population and used as a continuous measure of LSM intensity. LSM included larviciding and environmental management; modalities were not differentiated in the models.

### Data processing and management

Lags of 0–3 months were generated for time-varying climatic and environmental variables based on biological plausibility and prior evidence [10, 17]. Candidate lag structures were compared using DIC and WAIC. Missing covariate data affected < 2% of district months for any given variable, primarily due to occasional gaps in satellite data (Supplementary Fig S1). Missing covariate values were imputed using predictive mean matching with the *mice* package in R, after confirming that imputed and observed distributions were similar. Outcome data were not imputed.

Multicollinearity was assessed using variance inflation factors (VIFs) derived from linear approximations; all variables retained in the final models had VIF < 5.

Overdispersion was evident (variance greatly exceeding the mean), and negative binomial models had substantially better DIC/WAIC than Poisson models, so the negative binomial distribution was adopted.

### Statistical analysis

#### Model family and likelihood

Let ${Y}_{it}$ Confirmed malaria cases in the district $i$ at month $t$. We assumed a negative binomial distribution to account for overdispersion:

$${Y}_{it}\sim {\mathrm{NegBin}}({\mu }_{it},\kappa )$$

With mean ${\mu }_{it}$ and overdispersion parameter $\kappa$ (parameterization stated explicitly in Supplementary Methods to ensure reproducibility).

#### Linear predictor (primary spatiotemporal model)

$${\mathrm{log}}\left( {\mu_{it} } \right) = {\mathrm{log}}\left( {E_{it} } \right) + \beta_{0} + \mathop \sum \limits_{k} \beta_{k} X_{kit} + b_{i} + \delta_{t} + s_{m\left( t \right)}$$

where: $\mathrm{log}({E}_{it})$ is the population offset, ${X}_{kit}$ are covariates (climate/environment/interventions), ${b}_{i}$ is the spatial random effect (BYM2), ${\delta }_{t}$ is the temporal random effect (AR(1)), ${s}_{m(t)}$ is an explicit seasonal term for month-of-year.

Seasonality: we modeled seasonality using a cyclic random effect on month-of-year (12 levels) specified as cyclic RW1 (or equivalently, Fourier terms; results compared in sensitivity analyses). This reduces the risk that climate covariates act as proxies for unmodeled seasonality.

#### Spatial random effects: BYM2 model

Spatial structure was modeled using BYM2 [35, 36]; with an adjacency matrix defined by shared district borders (queen contiguity in the primary model; rook in sensitivity analysis). BYM2 combines structured (CAR) and unstructured (id) components with scaling.

$${b}_{i}=\frac{1}{{\tau }_{b}}\left(\sqrt{\rho }{\hspace{0.17em}}{u}_{i}^{*}+\sqrt{1-\rho }{\hspace{0.17em}}{v}_{i}\right),$$

where ${u}_{i}^{*}$ is the scaled intrinsic conditional autoregressive (CAR) component (structured spatial effect), ${v}_{i}$ is an unstructured i.i.d. component, $\rho \in [\mathrm{0,1}]$ is the mixing parameter indicating the proportion of spatial variance that is structured, and tau sub b is a precision parameter.

The inclusion of district-specific spatial random effects (BYM2) partially controls for confounding by indication, as these effects capture time-invariant unobserved district characteristics, including baseline endemicity that determines intervention targeting, thereby allowing intervention effects to be estimated from within-district (or within-neighborhood) contrasts between protected and unprotected periods.

#### Temporal random effects: AR (1) process

Temporal autocorrelation was modelled as a first-order autoregressive process:

$${\delta }_{t}=\phi {\hspace{0.17em}}{\delta }_{t-1}+{\eta }_{t},{\eta }_{t}\sim N(0,{\sigma }_{\delta }^{2}),\mid \phi \mid <1,$$

With ${\delta }_{1}\sim N\left(0,{\sigma }_{\delta }^{2}/(1-{\phi }^{2})\right)$. Here, $\phi$ captures persistence in residual risk over time.

We fitted separate models for: (i) total malaria cases; (ii) P. falciparum cases; and (iii) P. vivax cases.

We also fitted a district fixed-effects sensitivity model (Supplementary Table S2) in which each district has its own intercept, such that intervention effects are identified purely from *within-district temporal variation* in intervention coverage.

#### Spatiotemporal interaction (added sensitivity model)

To allow districts to deviate from the region-wide temporal pattern (important during localized outbreaks/resurgence), we fit a sensitivity model including a district-by-time interaction term:

- ${\gamma }_{it}\sim {\mathrm{id}}$ by district-month (Type I) or
- district-specific RW1 over time (Type II),
- and compared fit and impact on key covariates/hotspots

#### Climate effect decomposition (between vs within)

To distinguish geographic ecological gradients from within-district climate variability, we decomposed time-varying climate covariates:

$$X_{it} \, = \,\overline{X}_{i} \, + \,\left( {X_{it} \, - \,\overline{X}_{i} } \right)$$

Estimating separate coefficients for:

- Between-district (spatial) effect $\overline{X}_{i}$
- Within-district (temporal deviation) effect $\left( {X_{it} \, - \,\overline{X}_{i} } \right)$

#### Non-linearity (correction)

Because temperature and rainfall effects may be non-linear, we assessed non-linearity using penalized splines (second-order random walk) for key climate covariates in sensitivity analyses, comparing WAIC and examining whether linear IRRs masked threshold/optimum patterns.

### Prior specification and estimation

The study used penalized complexity (PC) priors for random-effect hyperparameters [37] following the BYM2 parameterization [35].

Spatial SD (${\sigma }_{b}$): PC before $\mathrm{Pr}({\sigma }_{b}>1)=0.01$, assigning low probability to very large spatial variability.

Spatial mixing parameter ($\rho$): PC prior with median 0.5, giving equal prior weight to structured and unstructured components.

Temporal AR (1) coefficient ($\phi$): PC prior centered at 0, penalizing strong temporal dependence, with most prior mass within $\mid \phi \mid <0.9$.

Dispersion ($\theta$): Weakly informative PC prior on $\mathrm{log}(\theta )$ to avoid overfitting.

Fixed-effect coefficients ${\beta }_{k}$ were assigned vague Gaussian priors, ${\beta }_{k}\sim N(\mathrm{0,100})$ (precision 0.01). Models were fitted using INLA for latent Gaussian models [38], implemented in the R-INLA package.

### Model building and sensitivity analyses

We followed a stepwise model-building strategy:

1. Baseline model: offset + BYM2 spatial random effects + AR(1) temporal random effects.
2. Climate model: baseline model + lagged rainfall and temperature.
3. Environment model: climate model + NDVI and elevation.
4. Full model: environment model + ITN ownership, IRS protection, and LSM intensity.

The final model for each outcome was selected based on the lowest DIC, WAIC, and epidemiological plausibility. Differences > 10 in DIC or WAIC were considered strong evidence favoring the model with the lower value.

### Variance decomposition

We quantified the relative contributions of fixed effects (climate, environment, and interventions), spatial random effects, and temporal random effects using marginal and conditional coefficients of determination (R^2^) for hierarchical models [39, 40]. Marginal R^2^ represents variance explained by fixed effects alone, while conditional R^2^ represents variance explained by both fixed and random effects [39]. For INLA-based spatiotemporal models, these were computed as:

$$R_{{{\mathrm{marginal}}}}^{2} = \frac{{{\mathrm{Var}}\left( {\hat{\mu }_{{{\mathrm{fixed}}}} } \right)}}{{{\mathrm{Var}}\left( {\hat{\mu }_{{{\mathrm{fixed}}}} } \right) + {\mathrm{Var}}\left( {b_{i} } \right) + {\mathrm{Var}}\left( {\delta_{t} } \right) + {\mathrm{Var}}\left( \varepsilon \right)}}$$

$$R_{{{\mathrm{conditional}}}}^{2} = \frac{{{\mathrm{Var}}\left( {\hat{\mu }_{{{\mathrm{fixed}}}} } \right) + {\mathrm{Var}}\left( {b_{i} } \right) + {\mathrm{Var}}\left( {\delta_{t} } \right)}}{{{\mathrm{Var}}\left( {\hat{\mu }_{{{\mathrm{fixed}}}} } \right) + {\mathrm{Var}}\left( {b_{i} } \right) + {\mathrm{Var}}\left( {\delta_{t} } \right) + {\mathrm{Var}}\left( \varepsilon \right)}}$$

where ${\mathrm{Var}}({b}_{i})$ and ${\mathrm{Var}}({\delta }_{t})$ denote spatial and temporal random-effect variances, respectively, and the residual variance was approximated as $1/\theta$ for the negative binomial model [41]. The relative contribution of covariate groups was assessed by sequential omission and changes in marginal R^2^ [42].

### Spatial and temporal decomposition of climate effects

To separate spatial (between-district) from temporal (within-district) climate effects, time-varying climate covariates were decomposed into district-specific means and monthly deviations from those means [43–46]:

$$X_{it} \, = \,\overline{X}_{i} \, + \,\left( {X_{it} \, - \,\overline{X}_{i} } \right)$$

Separate coefficients were estimated for $\overline{X}_{i}$, representing long-term spatial associations, and for $X_{it} \, - \,\overline{X}_{i}$, representing short-term temporal associations. This approach distinguishes ecological spatial effects from temporal climate variability influencing malaria transmission [47, 48].

### Temporal trend analyses

Temporal climate trends were evaluated using linear regression of monthly regional mean maximum temperature on time (months since January 2018) [49]. District-specific linear trends in maximum temperature and log malaria incidence were estimated using ordinary least squares regression [49], and associations between temperature and incidence trends were assessed using Spearman correlation [50].

### Sensitivity analyses, diagnostics, and mapping

Sensitivity analyses examined alternative penalized complexity priors for spatial and temporal random effects [51], different assumptions for indoor residual spraying effectiveness (4 and 8 months), and alternative spatial adjacency structures [36]. Model fit was assessed using posterior predictive checks, and residual spatial autocorrelation was evaluated using Moran’s I [52]. Posterior mean relative risks and residual spatial effects were mapped to identify unexplained spatial patterns [53].

### Addressing confounding by indication

We employed three complementary approaches:

BYM2 spatial random effects capture time-invariant district characteristics, including baseline endemicity that determines intervention targeting, allowing effects to be estimated from within-neighborhood contrasts.

District fixed-effects sensitivity model estimates intervention effects purely from within-district temporal variation, eliminating all between-district confounding.

Stratified analyses restricting to districts that received each intervention, testing whether effects persist when estimated only among recipients.

### Hotspot identification

Persistent malaria hotspots were identified using exceedance probabilities of the spatial random effects [54]. Districts with a posterior probability $P({\mathrm{RR}}>1.25\mid {\mathrm{data}})>0.75$ were classified as persistent hotspots, indicating elevated risk not explained by measured covariates [54, 55].

### Software

Analyses were conducted in R (version 4.5.2). Key packages: Spatial data were processed using *sf* and *terra*; plots were produced with *ggplot2* and *tmap*.

## Results

### Descriptive epidemiology of malaria resurgence

From January 2018 to December 2024, health facilities in 166 districts reported 5,746,571 confirmed malaria cases. P. falciparum comprised 3,696,996 cases (64.3%), while P. vivax accounted for 2,049,575 cases (35.7%).

**Fig. 3:**
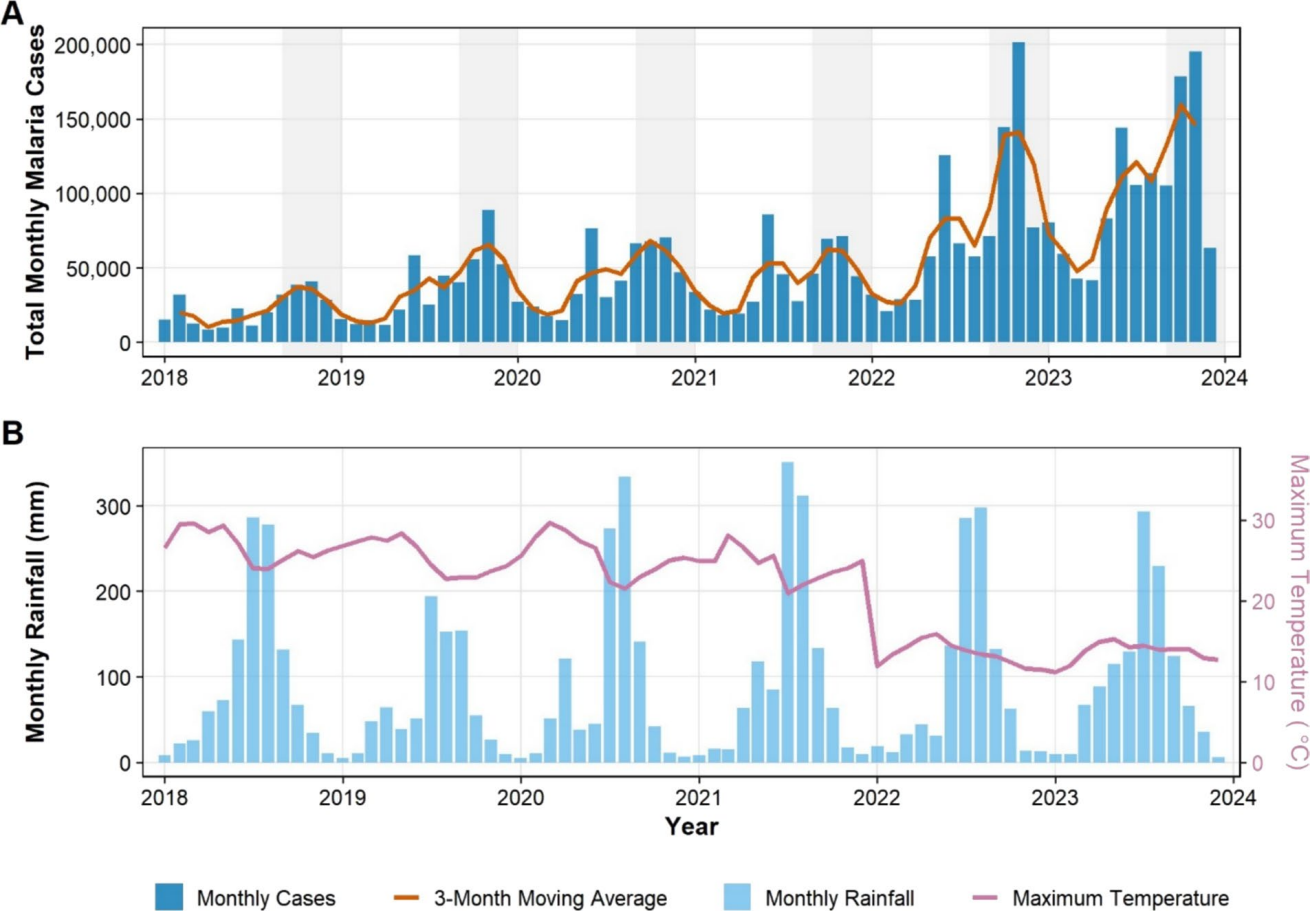
Temporal trends in malaria cases and climatic variables, Amhara Region, 2018–2024. **A** Monthly total malaria cases (bars) with 3-month moving average (red line), showing strong seasonality and an acceleration in incidence from 2022 onwards. Shaded areas indicate the main post-rainy transmission season (September–November). **B** Corresponding monthly cumulative rainfall (blue bars) and mean maximum temperature (red line), illustrating interannual climatic variability during the study period

Mean monthly incidence rose 5.5-fold, from 1.19 cases per 1,000 population in 2018 to 6.53 per 1,000 in 2024 (Table 1). This resurgence intensified after 2022 (Fig. 3A).

**Table 1:** Annual mean monthly malaria incidence (cases per 1,000 population), averaged across study districts (n = 166)

Year	Total incidence	P. falciparum	P. vivax
2018	1.19	0.88	0.31
2019	1.74	1.35	0.39
2020	1.89	1.44	0.45
2021	1.76	1.30	0.46
2022	3.09	2.10	0.99
2023	4.03	2.44	1.59
2024	6.53	4.09	2.44

### Climate and intervention trends

During this period, regional mean maximum temperature slightly declined from 30.1 °C in 2018 to 29.6 °C in 2024, following a linear trend of − 0.09 °C/year (95% CI − 0.15 to − 0.03; *p* = 0.004) (Fig. 3B). Rainfall exhibited high interannual variability without a significant linear trend (*p* = 0.31).

Across 13,944 district-months, mean ITN ownership stood at 65.2% (SD 18.5%). IRS protection covered only 21.5% of district-months within the 6-month post-spray window (Table 2, Fig. 3B).​

**Table 2:** Descriptive Statistics of Variables, Amhara Region (n = 13,944 district-months; 2018–2024)

Variable	Unit	Mean	Median	SD	Min	Max
Outcomes						
Total malaria cases	Count/district-month	412.1	185	679.5	0	9,871
P. falciparum cases	Count/district-month	265.1	98	478.2	0	7,234
P. vivax cases	Count/district-month	147.0	52	246.1	0	3,108
Climate/Environment						
Max temperature (0 lag)	°C	29.8	29.5	3.1	22.1	38.9
Rainfall (0 lag)	mm/month	168.3	115.6	104.8	0	589.4
NDVI (0 lag)	Unitless	0.45	0.46	0.16	0.11	0.88
Elevation	Meters	1,845	1,798	652	895	3,120
Interventions						
ITN ownership	% households	65.2	67.5	18.5	18	98
IRS protection	Binary (0/1)	0.215	0	0.41	0	1
LSM intensity	m^2^/1,000 pop	9.2	0	25.1	0	450.5

Climate variables shown at 0-lag; final models use 1-month lag for max temperature, 2-month lag for rainfall, 1-month lag for NDVI

### Bivariate temporal associations with climate and environment

**Fig. 4:**
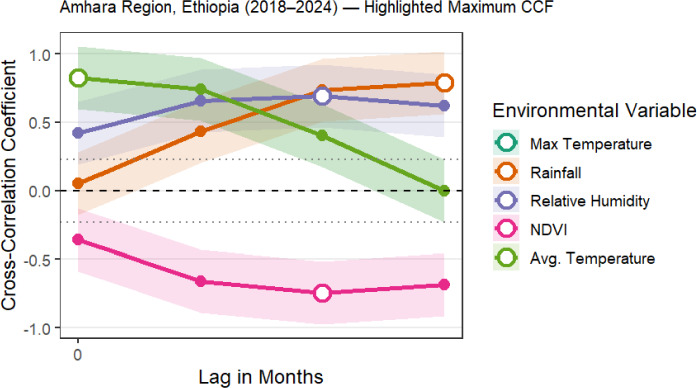
Standardised coefficients from univariable lagged models for selected climate and environmental covariates

Univariable models (Table 3; Fig. 4) showed that malaria incidence was positively associated with temperature and rainfall. Maximum temperature coefficients peaked at a 1-month lag (standardized IRR 1.18 per SD, equivalent to 1.18 per 3.1 °C increase). Rainfall coefficients peaked at a 2-month lag (IRR 1.15 per SD, equivalent to 1.15 per 104.8 mm increase).

**Table 3:** Univariable Lagged Associations with Total Malaria Incidence, Amhara Region (2018–2024)

Lag (months)	Rainfall	Mean temp	Max temp	Min temp	NDVI	Rel. humidity	Soil moisture
0	1.07	1.07	1.12	1.24	–0.11	1.13	1.10
1	1.10	1.09	**1.18**	1.25	–0.10	1.15	1.14
2	**1.15**	1.11	1.16	1.27	–0.10	1.15	1.16
3	1.13	1.13	1.14	1.28	–0.09	1.12	1.14

Values are standardized coefficients (IRR per 1 SD increase) from separate univariable negative binomial models with offset for district population, no random effects. NDVI coefficients are negative; all others are positive. Bold indicates lag selected for multivariable models

### Climate and environmental associations

**Fig. 5:**
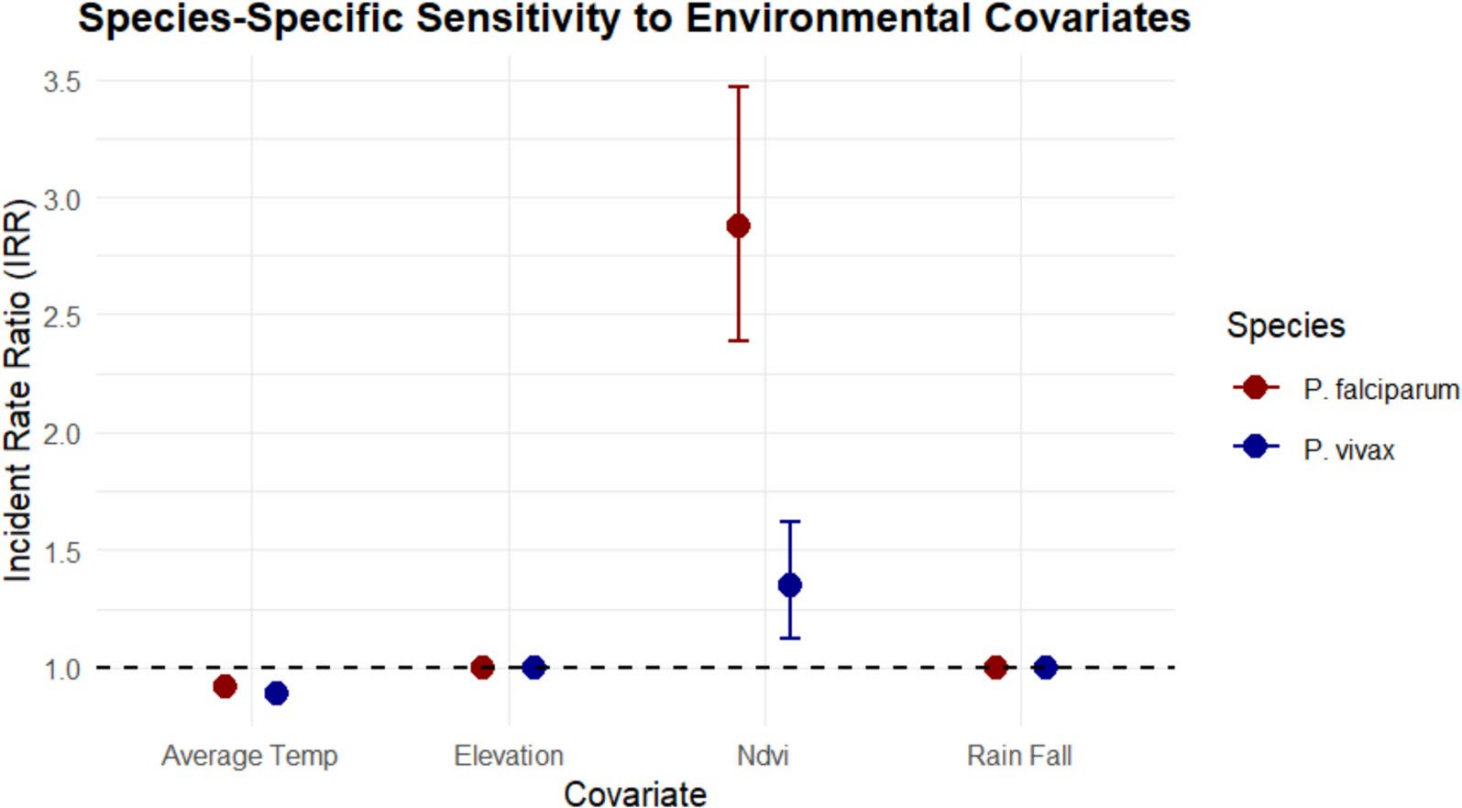
Species-specific sensitivity of malaria incidence to climate and environmental covariates

In fully adjusted Bayesian spatiotemporal models, a 1-SD (3.1 °C) increase in lagged maximum temperature associated with 15% higher total malaria incidence (IRR 1.15; 95% CrI 1.11–1.19), with stronger effects for *P. falciparum* (IRR 1.21; 95% CrI 1.16–1.26) than *P. vivax* (IRR 1.08; 95% CrI 1.03–1.13) (Table 4, Fig. 5). Lagged rainfall (2-month, 1-SD = 104.8 mm) linked to 9% higher incidence (IRR 1.09; 95% CrI 1.06–1.12).

**Table 4:** Fixed effects from Bayesian spatiotemporal models, Amhara Region (2018–2024)

Covariate	Scale	Total IRR (95% CrI)	*P. falciparum* IRR (95% CrI)	*P. vivax* IRR (95% CrI)
Environmental				
Rainfall (2-month lag)	1 SD (104.8 mm)	1.09 (1.06–1.12)	1.12 (1.08–1.16)	1.05 (1.01–1.09)
Max temperature (1-month lag)	1 SD (3.1 °C)	1.15 (1.11–1.19)	1.21 (1.16–1.26)	1.08 (1.03–1.13)
NDVI (1-month lag)	1 SD (0.16 units)	1.06 (1.02–1.10)	–	–
Elevation	per 100 m	0.91 (0.89–0.93)	0.88 (0.85–0.91)	0.94 (0.91–0.97)
Interventions				
IRS protection	6-month window	0.82 (0.78–0.86)	0.79 (0.74–0.84)	0.87 (0.82–0.92)
ITN ownership	per + 10 pp	0.94 (0.91–0.97)	–	–
LSM intensity	per + 1,000 m^2^/1,000 pop	0.98 (0.96–0.99)	–	–

*IRR* incidence rate ratio, *CrI* credible interval

"—" denotes covariates not retained in species-specific models. Models included BYM2 spatial and AR (1) temporal random effects with population offset. IRRs adjusted for covariates and random effects

Elevation proved protective: each 100-m rise associated with 9% lower total risk (IRR 0.91; 95% CrI 0.89–0.93), more so for *P. falciparum* (IRR 0.88; 95% CrI 0.85–0.91).

### Intervention associations

IRS during the 6-month protection window was associated with 18% lower total incidence (IRR 0.82; 95% CrI 0.78–0.86), stronger against *P. falciparum* (IRR 0.79) than *P. vivax* (IRR 0.87). ITN ownership reduced risk by 6% per 10 percentage-point rise (IRR 0.94; 95% CrI 0.91–0.97); LSM showed modest protection (IRR 0.98; 95% CrI 0.96–0.99) (Table 5). Stratified, fixed-effects, and pre-post analyses confirmed protective effects, ruling out major confounding by high-burden targeting (supplementary file: Table S3, Fig. S3).

**Table 5:** Intervention effects in all districts versus intervention-recipient districts, Amhara Region, 2018–2024

Intervention	Population	IRR	95% CrI	Districts	Months
IRS (6-month window)	All districts (Primary)	0.82	0.78–0.86	166	13,944
	Districts receiving 1 campaign	0.79	0.74–0.84	89	7,476
ITN ownership (+ 10 pp)	All districts (Primary)	0.94	0.91–0.97	166	13,944
	Districts with 1 year 50% coverage	0.93	0.89–0.96	158	13,272
LSM intensity (+ 1 unit)	All districts (Primary)	0.98	0.96–0.99	166	13,944
	Districts implementing LSM	0.97	0.94–0.99	47	3,948

### Pre-post comparison in IRS recipient districts

In 23 districts that initiated IRS for the first time during 2019–2022, mean monthly incidence declined by 28% (95% CI 19–36%) in the six months following the first spray compared to the same calendar months in the prior year (Supplementary Fig. S2), providing within-district quasi-experimental evidence of IRS effectiveness independent of spatial targeting.

### Variance decomposition (explained vs unexplained variability)

The full model explained 82.0% of district-month variance (conditional R^2^ = 0.82; 95% CrI 79.3–84.5), with fixed effects at 34.2% (climate/environment 26.2%; interventions 8.0%) and random effects at 47.8% (temporal 28.9%; spatial 18.9%) (Table 6). Residual unexplained variance was 18.0% (95% CrI 15.5–20.7) Table 6.

**Table 6:** Variance decomposition for the total malaria spatiotemporal model, Amhara Region, 2018–2024

Component	% Variance explained	95% CrI
Fixed effects (total)	34.2	30.8–37.9
Temperature + Elevation	18.3	15.2–21.7
Rainfall	7.9	6.0–10.2
Interventions	8.0	6.1–10.3
Random effects (total)	47.8	44.1–51.6
Temporal AR(1)	28.9	25.7–32.4
Spatial BYM2	18.9	16.0–22.1
Total explained (R^2^)	82.0	79.3–84.5
Residual	18.0	15.5–20.7

Marginal R^2^ (34.2%) represents variance explained by measured covariates (climate, environment, interventions) alone. Random effects (47.8%) capture structured spatiotemporal variation NOT explained by measured covariate likely reflecting unmeasured drivers such as conflict, insecticide resistance, and vector ecology. Total variance NOT explained by measured covariates = 47.8% + 18.0% = 65.8%. Conditional R^2^ (82.0%) represents total variance explained by fixed + random effects combined

*CrI* credible interval

### Temporal trend in regional mean temperature

Regional mean maximum temperature (averaged across all 166 districts each month) showed a slight declining trend over the study period: –0.09 °C per year (95% CI –0.15 to –0.03 °C; p = 0.004; linear regression), from a mean of 30.1 °C in 2018 to 29.6 °C in 2024 (Supplementary Fig. S4). This indicates that the fivefold increase in malaria incidence occurred during a period of slight regional cooling, not warming, at the aggregated regional scale. Time series of regional mean monthly maximum temperature (2018–2024) with linear trend line (slope = –0.09 °C/year, p = 0.004).

### Climate Effect Decomposition

Spatial (between-district) climate effects exceeded temporal (within-district) ones: max temperature spatial IRR 1.42 (95% CrI 1.35–1.49) vs. temporal 1.06 (1.02–1.10); rainfall 1.18 vs. 1.08. No district-level link tied warming trends to incidence rises (Spearman ρ = 0.08; *p* = 0.29) supplementary Fig. 4.

### Spatiotemporal Random Effects

Temporal autocorrelation was substantial, with the AR(1) coefficient ϕ = 0.74 (95% CrI 0.70–0.78) for total malaria. Temporal persistence was higher for *P. vivax* (ϕ = 0.79; 95% CrI 0.75–0.83) than for *P. falciparum* (ϕ = 0.68; 95% CrI 0.64–0.72), suggesting greater residual month-to-month persistence in *P. vivax* transmission dynamics (Table 7).

**Table 7:** Spatiotemporal Random Effect Parameters, Amhara Region, 2018 to 2024

Parameter	Total Malaria (95% CrI)	*P. falciparum* (95% CrI)	*P. vivax* (95% CrI)
AR(1) coefficient (ϕ)	0.74 (0.70–0.78)	0.68 (0.64–0.72)	0.79 (0.75–0.83)
BYM2 mixing (ρ)	0.88 (0.80–0.96)	0.85 (0.79–0.90)	0.77 (0.70–0.83)
Spatial SD (σ_b)	0.42 (0.35–0.50)	0.48 (0.40–0.57)	0.34 (0.28–0.41)
Temporal SD (σ_δ)	0.31 (0.26–0.37)	0.29 (0.24–0.35)	0.35 (0.29–0.42)

AR(1) coefficient indicates the strength of residual temporal autocorrelation. BYM2 mixing parameter indicates the proportion of spatial variance that is structured (shared among neighbors) vs. unstructured (district-specific). Higher ϕ for *P. vivax* suggests greater temporal persistence, possibly reflecting relapse dynamics

Spatial Heterogeneity and Hotspot Identification

Spatial variance was predominantly structured, with BYM2 mixing parameter ρ = 0.88 (95% CrI 0.80–0.96), indicating that most unexplained spatial variability was shared among neighboring districts rather than being district-specific.

**Fig. 6:**
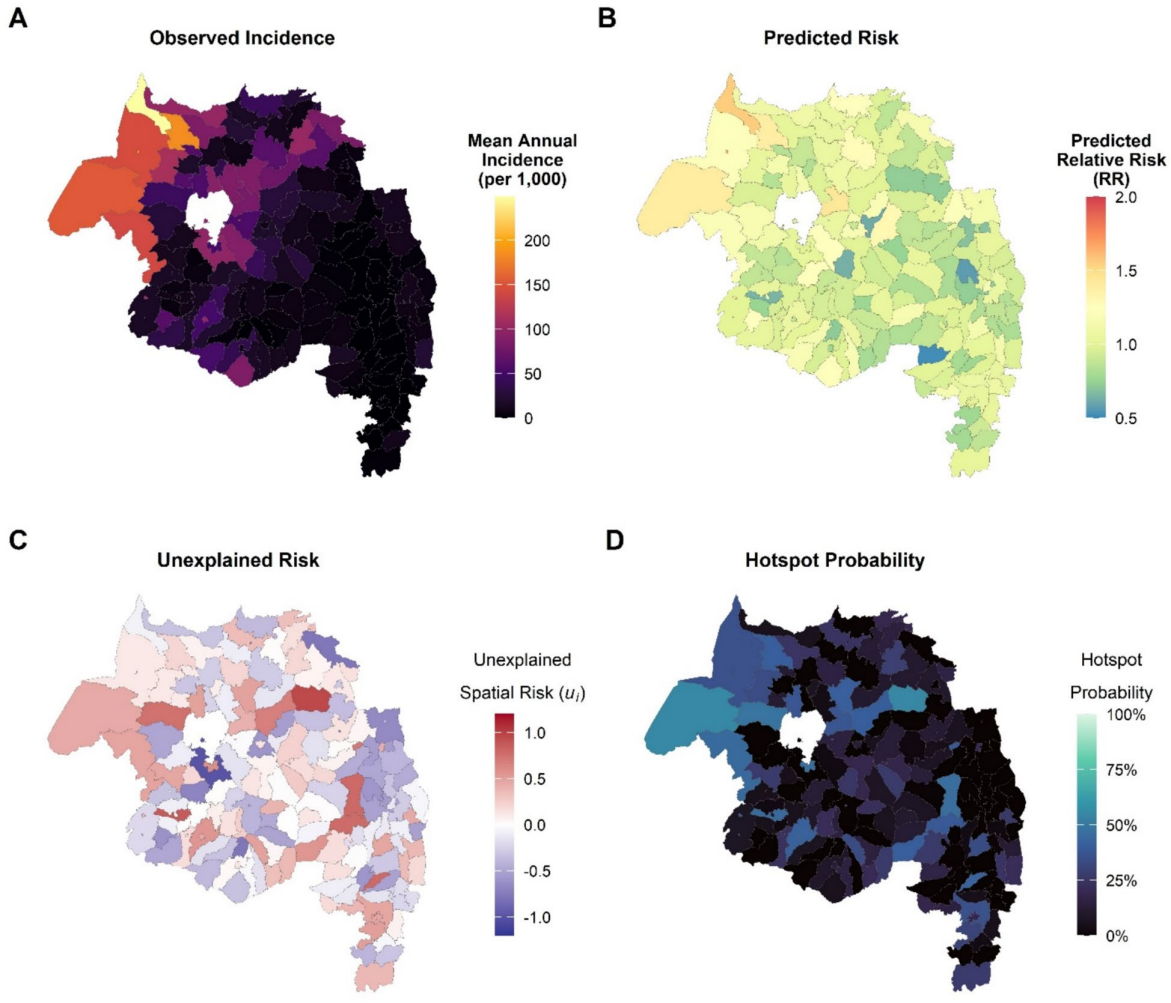
Spatial heterogeneity and hotspots of malaria risk in Amhara Region, 2018–2024. **A** Observed mean annual total malaria incidence per 1,000 population by district. **B** Posterior mean relative risk (RR) of total malaria from the full Bayesian model. **C** Posterior mean of the spatial random effect (residual RR), representing unexplained spatial variation after adjustment for covariates. **D** Exceedance probability map P(residual RR > 1.25), highlighting persistent hotspots (P > 0.75) in low-elevation western border districts, including Metema, Quara, West Armachiho, Tach Armachiho, Tegede and Mirab Belessa

Maps of observed mean annual incidence and model-predicted RR demonstrated intense spatial heterogeneity (Fig. 6). To identify persistent hotspots beyond what was explained by covariates, the posterior mean of the spatial random effect (residual RR) and its exceedance probability were examined.

Districts with a posterior probability > 0.75 of having a residual RR > 1.25 were classified as persistent hotspots. These included Metema, Quara, West Armachiho, Tach Armachiho, Tegede, Mirab Belessa, and adjacent low-elevation western border districts (Fig. 6D). These districts are characterised by extensive agriculture, lower elevation, and substantial cross-border and seasonal labour mobility.

### Seasonal and temporal dynamics

**Fig. 7:**
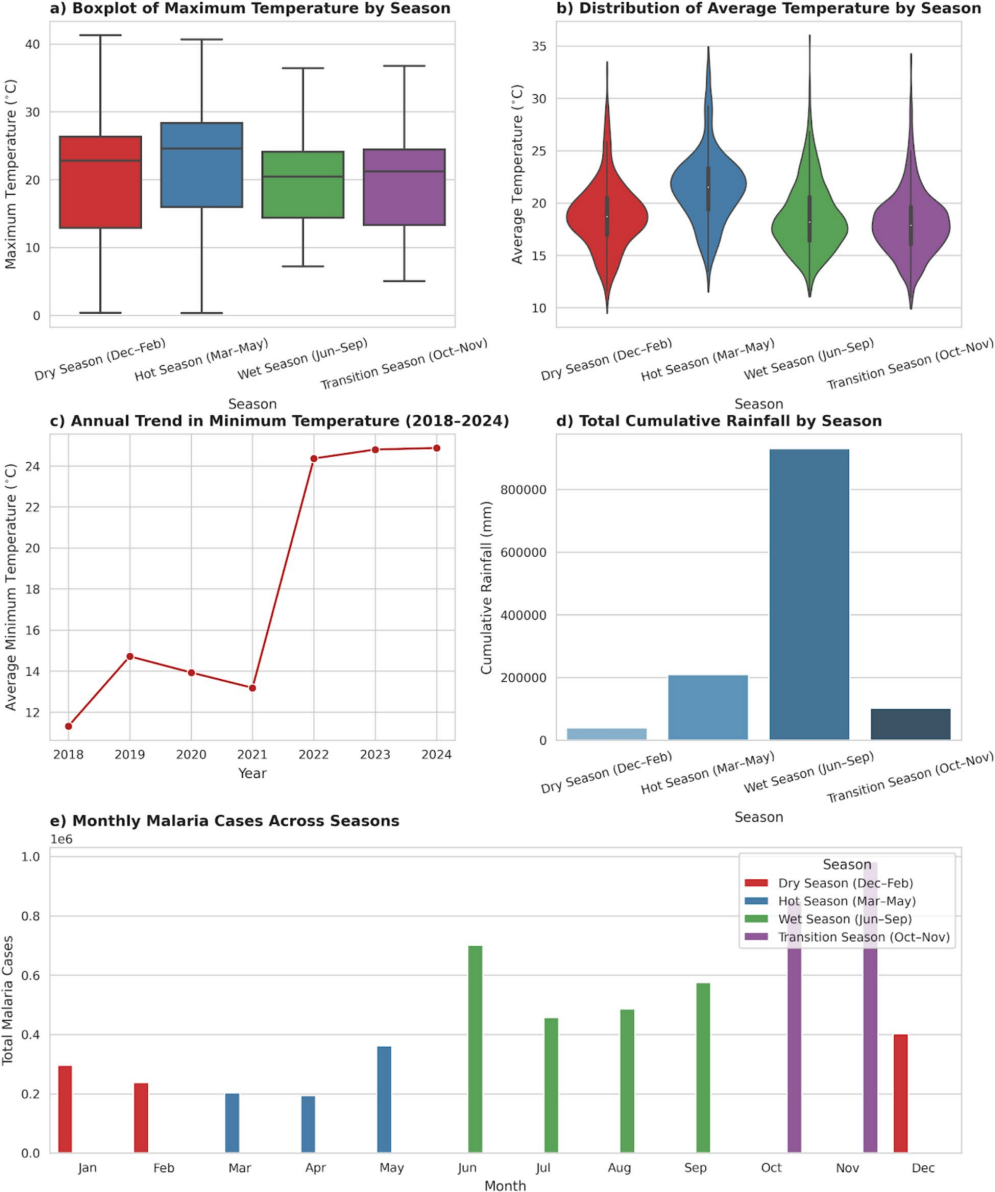
Seasonal and interannual patterns of temperature, rainfall and malaria incidence, Amhara Region, 2018–2024. **A** Boxplots of maximum temperature by meteorological season (DJF, MAM, JJA, SON). **B** Boxplots of mean temperature by season. **C** Annual trend in minimum temperature. **D** Seasonal rainfall totals by month, aggregated over the study period. **E** Seasonal pattern of monthly malaria cases (all years aggregated), coloured by season, showing peak incidence in the post-rainy transition months (September–November)

Seasonal patterns of climate and malaria incidence are shown in Fig. 7. Rainfall peaked from June to September, while malaria incidence peaked 1–2 months later, during the post-rainy season (September–November). Maximum temperatures were highest in the pre-rainy season (March–May), coinciding with the early rise in cases. These patterns are consistent with rainfall-driven increases in vector breeding followed by temperature-dependent parasite development.

### Model performance and diagnostics

For the total malaria model, WAIC and DIC were 3,612 and 3,598, respectively, indicating good predictive performance and parsimony. A conditional pseudo-R^2^ of 0.82 suggested that fixed and random effects jointly explained 82% of the variance in district-month incidence. The root mean square error (RMSE) on the log scale was 0.09, indicating close agreement between observed and predicted values.

Standardised residuals showed no significant residual spatial autocorrelation (Moran’s I = 0.04; p = 0.21), suggesting that spatial structure was adequately captured. Posterior predictive checks indicated good agreement between observed and replicated distributions of counts (Supplementary Fig. S4; Table 8).

**Table 8:** Model performance metrics for the total malaria model, Amhara Region (2018–2024)

Metric / parameter	Value	Interpretation
WAIC	3,612	Lower values indicate better predictive fit
DIC	3,598	Lower values indicate a good fit with parsimony
Conditional R^2^	0.82	82% of variance explained by fixed + random effects
RMSE (log-scale)	0.09	Close agreement between predicted and observed
Moran’s I (residuals)	0.04 (p = 0.21)	No significant residual spatial autocorrelation
AR(1) coefficient (φ)	0.74	Strong temporal autocorrelation

## Discussion

The findings of this study extend our earlier descriptive work [14], which documented the phenomenon of malaria resurgence in Amhara through Joinpoint regression and spatial hotspot analysis. Whereas the previous study established 'what' happened, demonstrating the magnitude, temporal trajectory, and geographic expansion of the resurgence, the present Bayesian spatiotemporal analysis elucidates 'why' these patterns emerged by quantifying the associations between incidence trends and specific climatic, environmental, and programmatic covariates. This analytical progression from description to explanation provides the mechanistic understanding necessary for designing evidence-based intervention strategies.

This district-level Bayesian spatiotemporal analysis provides a detailed characterization of malaria resurgence in Amhara Region between 2018 and 2024. To our knowledge, it is the first study in Ethiopia to jointly assess climate, environmental context, intervention coverage, and species-specific transmission dynamics using district-month data within a unified BYM2–INLA framework.

A key finding is that climate incidence associations are predominantly spatial rather than temporal. The between-district temperature effect (IRR 1.42) was seven times larger than the within-district effect (IRR 1.06). This indicates that observed associations reflect geographic ecology transmission is inherently more intense in hot, wet lowlands rather than temporal warming driving the recent resurgence. This interpretation is strongly supported by the paradox that regional temperature declined during the study period (− 0.09 °C/year), while malaria incidence increased 5.5-fold. If temporal warming were driving resurgence, we would expect positive correlations between district-level temperature trends and incidence trends; instead, we found no association (ρ = 0.08).

The findings indicate that variability in temperature and rainfall is strongly associated with variability in malaria incidence across space and time in Amhara. A 3.1 °C increase in maximum temperature at a 1-month lag was associated with 15–21% higher incidence, and a 105-mm increase in rainfall at a 2-month lag with 9–12% higher incidence. These magnitudes are consistent with mechanistic understanding and empirical studies linking climate to *Anopheles* ecology and malaria risk [15–19, 56–59]. In particular, the stronger association of maximum temperature with *P. falciparum* supports the hypothesis that warmer conditions are especially favourable for *P. falciparum* transmission in highland-fringe environments [56, 58, 59]. Our use of fine-scale district-level monthly data improves upon earlier analyses that relied on coarser spatial or temporal resolutions.

Species-stratified models showed that *P. falciparum* incidence was more sensitive to contemporaneous climate, whereas *P. vivax* incidence exhibited stronger residual temporal dependence and a weaker decline with elevation. These patterns are consistent with parasite biology: *P. falciparum* transmission depends more directly on current vector–host contact [18, 60], whereas *P. vivax* can relapse months after primary infection due to liver-stage hypnozoites [34, 61]. At the same time, the AR (1) temporal component represents residual persistence in incidence and may also reflect unmeasured programmatic or behavioural factors. Operationally, climate-informed early warning systems and timely intensification of vector control may be particularly impactful for *P. falciparum*, while *P. vivax* elimination will require strategies that explicitly address relapse (e.g. radical cure with G6PD testing) and persistent foci at higher altitudes [34, 59–61].

Elevation emerged as a strong protective factor, particularly for *P. falciparum*, echoing evidence that *P. vivax* tolerates cooler temperatures and persists at higher altitudes [12, 13]. NDVI was modestly positively associated with total malaria, suggesting that vegetation and related land-use patterns (e.g., irrigated agriculture) may favor vector survival and transmission [34, 62]. These findings highlight the role of environmental and land-use change in shaping malaria risk.

All three vector control tools, IRS, ITNs, and LSM, retained protective associations. IRS was associated with 18–21% lower incidence overall and 21% lower *P. falciparum* incidence during protected months, consistent with field evaluations [21, 30]. However, temporal coverage was limited, with only about 22% of district-months under IRS protection, and implementation may have been misaligned with peak risk. High reported ITN ownership may conceal issues with net condition, pyrethroid resistance, and use; widespread resistance in *An. arabiensis* is documented in Amhara [21, 63]. LSM was low-intensity and highly localized, contributing only a small marginal effect at the regional scale.

These patterns suggest that tools remain effective but are not deployed optimally in space and time. In line with the WHO’s “High burden to high impact” initiative [25], a shift from uniform coverage to data-driven, targeted intervention packages is warranted, especially in high-risk districts and seasons identified by spatiotemporal models.

The variance decomposition reveals that despite including climate, environment, and intervention covariates, substantial variability remains unexplained. Fixed effects explained only 34% of the variance, while random effects captured an additional 48%, leaving 18% entirely unexplained. The large temporal random effect component (29%) indicates persistent month-to-month variation not captured by measured covariates.

This is consistent with important unmeasured factors that the reviewer appropriately highlighted: Conflict-related health system disruptions: Multiple Amhara zones experienced armed conflict since 2020, with documented facility closures and commodity stockouts [31]. These disruptions are not systematically captured in routine data. Insecticide resistance: Widespread pyrethroid resistance in *An. arabiensis* is documented in Amhara [21], potentially reducing ITN effectiveness despite high reported ownership. Vector ecological changes: Emergence of *An. stephensi* has been documented in Ethiopia [28, 30], potentially altering transmission dynamics. Population displacement: Conflict-driven population movement may have increased exposure in displaced populations and disrupted intervention coverage. Reporting changes: Although diagnostic protocols remained unchanged, surveillance system performance may have varied temporally. We explicitly acknowledge that our measured covariates cannot fully explain the observed resurgence, and caution against causal interpretation of the reported associations**.**

The identification of a small number of persistent hotspots, primarily low-elevation western border districts such as Metema, Quara, West Armachiho, Tach Armachiho, Tegede, and Mirab Belessa, is a key operational insight. These districts have high residual RR after adjusting for climate, environment, and interventions, and they are characterised by intensive commercial agriculture and cross-border and seasonal labour migration [10, 17, 30], consistent with a source–sink dynamic. A “source reduction” strategy that concentrates next-generation IRS, high-quality ITNs, focal LSM, enhanced case management, and proactive case detection in these districts, coupled with cross-border collaboration with Sudan, is likely to yield substantial regional benefits.

From a methodological perspective, the use of posterior probabilities(P[RR > 1.25] > 0.75) to define hotspots moves beyond simple threshold-based incidence maps and provides a more robust, uncertainty-aware basis for prioritization. The highly structured spatial fraction (ρ = 0.88) emphasizes the need for spatially coherent strategies at the sub-regional scale rather than independent district-level planning. The combination of lagged climate effects and AR(1) temporal components provides a foundation for operational early warning systems.

## Strength and limitation

The study strengths include the use of multi-year district-month surveillance data across a period of documented resurgence; application of a Bayesian spatiotemporal framework that accommodates overdispersion, spatial dependence, and temporal autocorrelation; and the explicit decomposition of climate effects into spatial and temporal components to improve interpretability. The use of uncertainty-aware exceedance probabilities for identifying districts with elevated residual spatial risk also supports decision-making under uncertainty.

As an ecological study using aggregate district-level data, this analysis cannot establish causality or rule out important unmeasured confounding. We lacked explicit data on conflict exposure, health facility functionality, population displacement, vector species composition, and temporal changes in diagnostic capacity or reporting completeness. Our findings should therefore be interpreted as identifying spatial and temporal associations that can guide hypothesis generation and programmatic prioritization, rather than definitive causal attributions.

## Conclusion

Malaria resurgence in Amhara Region between 2018 and 2024 is consistent with increased climatic suitability, especially higher maximum temperatures, superimposed on incomplete and spatially mismatched deployment of effective vector control. The resurgence is characterized by climate-sensitive, lowland-dominated *P. falciparum* transmission; more temporally persistent *P. vivax* transmission is less closely tied to current weather, and a small set of persistent, low-elevation, cross-border hotspots that likely act as regional sources of infection.

To advance Ethiopia’s malaria elimination goals, programmes should move from uniform regional coverage to climate-informed, hotspot-focused, and species-tailored strategies. Priority actions include intensifying multi-pronged interventions in persistent hotspots, developing climate-triggered early warning systems, expanding *P. vivax* radical cure and relapse prevention, and integrating emerging vectors such as *An. stephensi* into surveillance and control, and alongside strengthened surveillance systems to identify and address unmeasured drivers of temporal surges.

## Supplementary Information

Additional file 1.

## Data Availability

The routine surveillance datasets analysed during the current study are available from the Amhara Public Health Institute (APHI) on reasonable request and subject to institutional data-sharing policies. Climatic and population datasets are publicly accessible from their respective repositories (see Supplementary Table S1). The analysis code is available from the corresponding author on reasonable request.

## Abbreviations

APHI: Amhara Public Health Institute
AR(1): Autoregressive of order 1
BYM2: Besag–York–Mollié 2
CAR: Conditional autoregressive
CHIRPS: Climate Hazards Group InfraRed Precipitation with Station data
CrI: Credible interval
CSA: Central Statistical Agency
DIC: Deviance information criterion
ERA5: ECMWF Reanalysis 5
INLA: Integrated Nested Laplace Approximation
IRS: Indoor residual spraying
ITN: Insecticide-treated net
LSM: Larval source management
NDVI: Normalized Difference Vegetation Index
*Pf*: *Plasmodium falciparum*
*Pv*: *Plasmodium vivax*
RDT: Rapid diagnostic test
RR: Relative risk
SRTM: Shuttle radar topography mission
WAIC: Widely applicable information criterion
