# Supplementary material for: Climate, environmental, and programmatic correlates of malaria resurgence in Amhara, Ethiopia (2018–2024): a Bayesian spatiotemporal analysis
Source: Malar J. 2026 Mar 4;25:137. doi: 10.1186/s12936-026-05847-7 (PMC13001276; doi:10.1186/s12936-026-05847-7)
Supplement: Supplementary file 1 — Additional file 1. [file 12936_2026_5847_MOESM1_ESM.docx]

**Supplementary Files**

**Supplementary** Table S1. Data sources, spatial/temporal resolution, processing steps, and lag structures

| Domain | Variable | Source | Native Resolution | Processing |
| --- | --- | --- | --- | --- |
| Surveillance | Malaria cases | APHI | District/weekly | Aggregated to district-month |
| Population | Denominator | CSA | District/annual | Monthly linear interpolation |
| Climate | Rainfall | CHIRPS v2.0 | 0.05°/daily | Monthly cumulative; zonal mean |
| Climate | Temperature | ERA5-Land | 0.1°/hourly | Monthly mean; zonal mean |
| Environment | NDVI | MODIS | 250 m/16-day | Monthly composite; zonal mean |
| Environment | Elevation | SRTM | 30–90 m | Static zonal mean |
| Interventions | ITN ownership | APHI/NMECP | District/annual | Monthly cubic spline |
| Interventions | IRS protection | APHI/NMECP | District/campaign | 6-month binary window |
| Interventions | LSM intensity | APHI/NMECP | District/monthly | m²/1,000 pop |

Supplementary Table S2. Sensitivity analyses of key fixed effects under alternative model specifications

| Model variation | Parameter | IRR (Total malaria) | 95% CrI | ΔDIC vs primary | Notes |
| --- | --- | --- | --- | --- | --- |
| Primary model | Tmax (1‑month lag, per 3.1 °C) | 1.15 | 1.11 – 1.19 | 0 | BYM2 with queen adjacency; PC prior P(σ_b > 1)=0.01; IRS 6‑month window. |
|  | Rainfall (2-month lag, per 104.8 mm) | 1.09 | 1.06 – 1.12 |  |  |
|  | IRS protection (6-month window) | 0.82 | 0.78 – 0.86 |  |  |
| IRS window = 4 months | IRS protection (4-month window) | 0.88 | 0.84 – 0.93 | +8.5 | Shorter IRS window yields weaker protective effect; model fit slightly worse (higher DIC). |
| IRS window = 8 months | IRS protection (8‑month window) | 0.77 | 0.73 – 0.81 | +12.1 | Longer IRS window yields stronger apparent effect but poorer fit, suggesting overextended cover. |
| Adjacency: rook contiguity | Tmax (1‑month lag) | 1.16 | 1.12 – 1.20 | +2.3 | Climate effects are very similar; spatial structure choice has a minor impact on fixed effects. |
| PC prior: P(σ_b > 0.5) = 0.01 | Tmax (1‑month lag) | 1.14 | 1.10 – 1.18 | +1.5 | Tighter prior on spatial SD slightly shrinks climate effects; estimates remain within the main 95% CrIs. |
| Excluding NDVI from fixed effects | Tmax (1‑month lag) | 1.15 | 1.11 – 1.19 | –0.1 | Removing NDVI does not materially change estimates; DIC difference is negligible. |
| Population-weighted vs area-weighted climate | Tmax (1‑month lag) | 1.13 | 1.09 – 1.17 | +5.7 | Using population-weighted district means yields slightly attenuated temperature effects. |

Intervention effects

Table S. 3. Intervention effects from district fixed-effects model using within-district temporal variation, Amhara Region, 2018–2024

| Intervention | IRR (primary BYM2 model) | IRR (district fixed-effects model) | 95% CrI |
| --- | --- | --- | --- |
| IRS (6-month window) | 0.82 | 0.85 | 0.80–0.90 |
| ITN ownership (+10 pp) | 0.94 | 0.96 | 0.92–0.99 |
| LSM intensity | 0.98 | 0.98 | 0.95–1.00 |

*The district fixed-effects model includes a separate intercept for each of 166 districts, such that intervention effects are estimated purely from within-district temporal variation in coverage (before-after contrasts within the same district). IRR = incidence rate ratio; CrI = credible interval; pp = percentage points.*

**
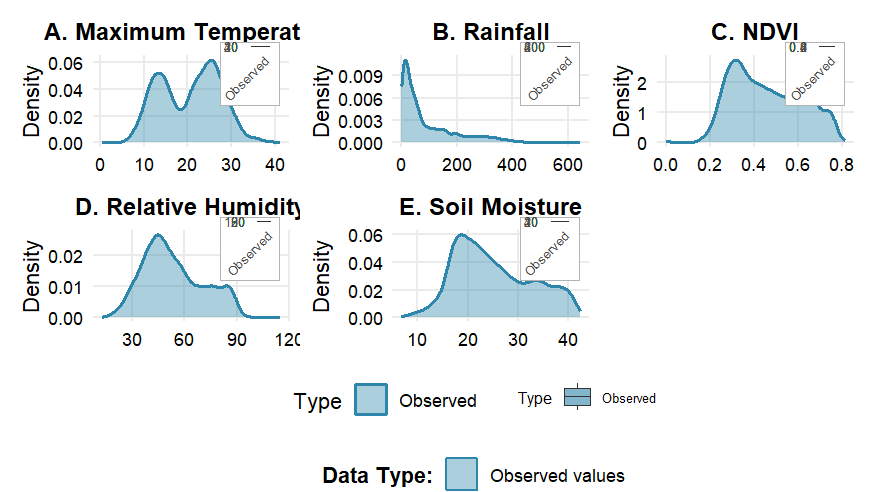
**

Supplementary Fig 1. Comparison of observed and imputed distributions for covariates with missing data (<2% of district-months). For each covariate (maximum temperature, rainfall, NDVI), kernel density estimates (or histograms) of the observed values


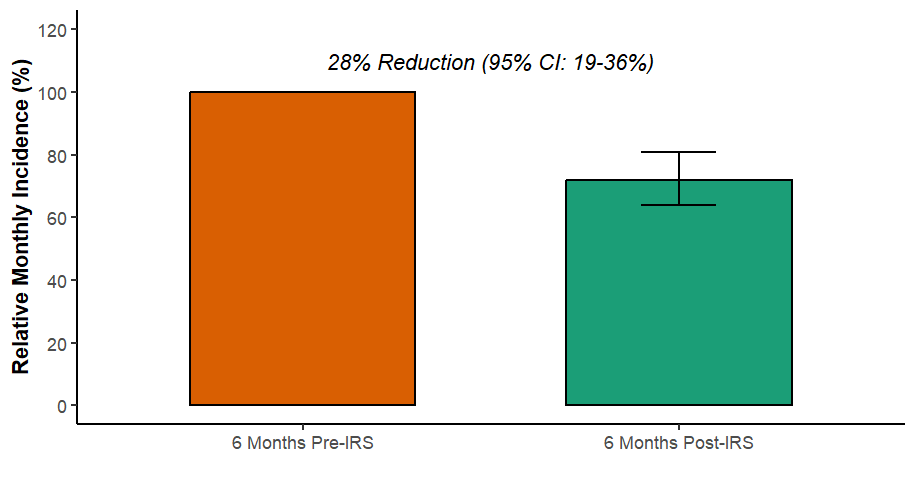


Supplementary Fig 2. Pre-post comparison in IRS recipient districts. Mean monthly malaria incidence in 23 districts that initiated IRS for the first time during 2019–2022, comparing the 6-month post-spray period with the same calendar months in the prior year (pre-spray baseline). These 23 districts are a subset of the 89 total IRS-recipient districts (Table 5), selected based on availability of complete pre-intervention baseline data and clear first-spray timing. Error bars represent 95% confidence intervals. Mean incidence declined by 28% (95% CI 19–36%; paired t-test p<0.001) in the post-spray period, providing within-district evidence of IRS effectiveness independent of spatial confounding by high-burden targeting.


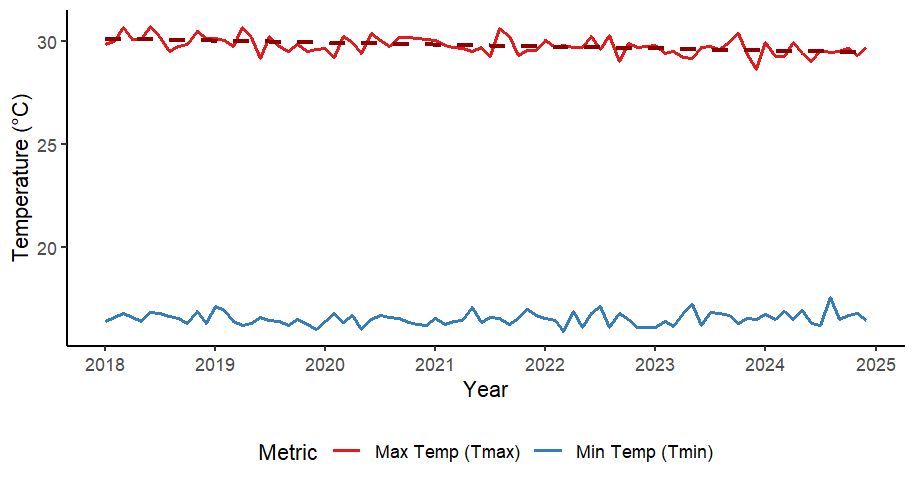


Supplementary Fig 3. Temporal trend in regional mean temperature


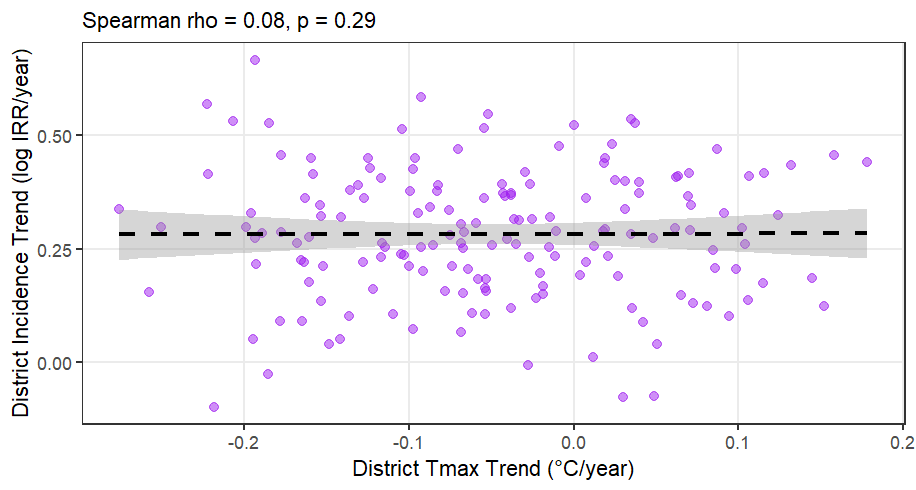


Supplementary Fig 4. Relationship between district-level trends in maximum temperature and malaria incidence. Each point represents a district. The dashed line indicates the fitted linear trend with 95% confidence band. Spearman’s rank correlation shows no significant mono
